# Effect of Ultraviolet C Irradiation on Isoflavone Concentrations in Different Cultivars of Soybean (*Glycine max*)

**DOI:** 10.3390/plants9081043

**Published:** 2020-08-16

**Authors:** Krishna Bahadur Karki, Awdhesh Kumar Mishra, Seong-Jin Choi, Kwang-Hyun Baek

**Affiliations:** 1 Research and Development Society, Kathmandu 4179, Nepal; kkarki2000@gmail.com; 2 Department of Biotechnology, Yeungnam University, Gyeongsan, Gyeongbuk 38541, Korea; awadhesh.biotech07@gmail.com; 3 Department of Biotechnology, Catholic University of Daegu, Gyeongsan 38430, Korea; sjchoi@cu.ac.kr

**Keywords:** *Glycine max*, UV-C, phytoestrogen, isoflavone, induction, irradiation

## Abstract

Phytoestrogens are naturally occurring plant polyphenolic compounds present in high concentrations in soybean products. Phytoestrogens are divided into three classes: lignans, isoflavones, and coumestans. Nine types of glycoside isoflavones and three types of aglycoside isoflavones are reported in soybean. Soy isoflavones can reduce the risk of a certain type of cancer, cardiovascular problems, osteoporosis, and menopausal symptoms. We irradiated the leaves of five cultivars of soybean with UV-C (260 nm) and determined the effect on concentrations of isoflavone compounds using liquid chromatography–mass spectrometry (LC–MS). Isoflavone concentrations were significantly higher following irradiation, particularly in the cultivar Daepung, which was selected as the best cultivar for high isoflavone induction with UV-C irradiation. Further experimentation with the cultivar Daepung revealed that 20 min UV-C irradiation was the best treatment for the induction of aglycone compounds, and 5 min with the dorsal surface facing the UV-C irradiation source was the best treatment for the induction of glycoside isoflavone compounds.

## 1. Introduction

The solar ultraviolet (UV) spectrum is a continuous spectrum of light that can be divided into three wavelength bands: UV-A (320–400 nm), UV-B (280–320 nm), and UV-C (200–280 nm). The negative effect of UV radiation for living organisms increases as wavelengths shorten. UV-A has a less detrimental effect and plays a significant role in plant photomorphogenesis. UV-B radiation is potentially harmful to living organisms and absorbed to some extent by the atmospheric ozone layer, which reduces its ability to reach the earth’s surface. UV-C radiation, which has a shorter wavelength, causes numerous injuries. It is often described as “germicidal UV” due to its ability to deactivate the DNA of a broad range of microorganisms [1]. The dose, which is computed as the multiplication of the intensity (Wm-2) and time of exposure (seconds), is expressed as radiant exposure (Jm-2). UV-C irradiation is well documented for its effect on plants, and light-emitting diodes have been used for this purpose [2,3,4]. A low dose of UV-C may activate acclimation responses in plants, including the activation of enzymatic and non-enzymatic defense systems [5,6,7,8]. High doses of UV-C radiation lead to an overproduction of reactive oxygen species (ROS) that results in oxidative stress, decreased cell viability, and cell death [9,10,11,12,13,14]. Application of low UV-C doses have been considered for commercial purposes as they can prevent pathogenic diseases and delay senescence during fruit and vegetable storage [15,16]. However, exposure to ultraviolet radiation can cause various alterations in plant metabolism and affect its biochemical activities [17,18,19].

Metabolites are compounds synthesized by plants for a specific function. Metabolites involved in growth and development are known as primary metabolites. Secondary metabolites are present incidentally, since they are not essential for plant life [20]. Although secondary metabolites are not essential for survival, they are involved in protection, competition, and species interactions. Secondary metabolites are specific to individual species [21]. Phytoestrogens are naturally occurring plant phenolic, non-steroidal compounds, which are classified as isoflavones, coumestans, and lignans. These compounds are present in high amounts in soy products (e.g., beans and tofu); plant lignans are found in legume grains, seeds, and vegetables [22]. Phytoestrogens are heterocyclic phenolic compounds with a structure similar to that of estrogenic steroids (mammalian endogenous estrogens). Therefore, these compounds show estrogen-like activity or weak antiestrogen-like properties [23]. Isoflavones show clear estrogenic activity, and these compounds are effective for estrogenic therapy [24,25]. In addition to isoflavonoids, flavonoids also exert estrogenic effects, but usually at considerably lower levels than those of isoflavonoids [26]. Moreover, some flavonoids, including kaempferol and quercetin, exhibit antiestrogenic activity [27], and several legumes are a source of these flavonoids [28,29].

**Figure 1:**
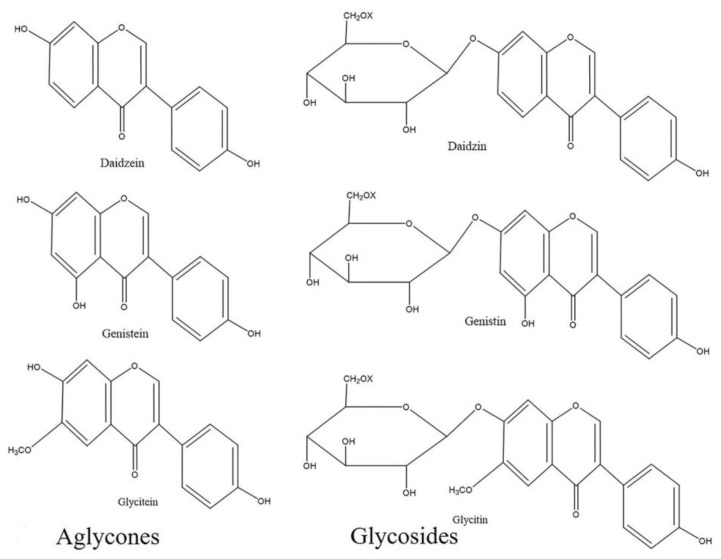
Chemical structure of soybean isoflavones. In soybeans, isoflavones are present in two isoforms: aglycones and glycosides. Release of the sugar moiety from glycosides often generates aglycones. Glycosides have three chemical isoforms: β-glucoside, acetylglucoside, and malonyl glucoside (X represents H, COCH_3_, and COCH_2_COOH in β-glucoside, acetylglucoside and malonylglucoside, respectively). Chemical structures were drawn using ChemDraw Standard 13.0 software (PerkinElmer, Waltham, MA, USA).

There are 12 isoflavone compounds in soybean (*Glycine max*) seeds including 9 types of glycosides (daidzin, malonyldaidzin, acetyldaidzin, glycitin, malonylglycitin, acetylglycitin, genistin, malonylgenistin, and acetylgenistin) and 3 types of aglycones (daidzein, genistein, and glycitein) (Figure 1) [25,30,31]. Other isoflavones are also present in legumes, such as biochanin-A and formononetin [32]. Genistein and daidzein are the predominant and most commonly investigated isoflavones in human nutrition. They can be obtained from soybeans and other legumes including peas, lentils, and beans [33,34]. Generally, soybean plants produce isoflavone compounds in response to environmental stresses. The plants produce different types of isoflavones for defense purposes, and some isoflavones function as antibiotics and antifungal compounds. In addition, some isoflavones play a vital role in cell regulation [35]. Soybeans consist of about two grams of isoflavones per kilogram of fresh weight [36].

Legumes are present in almost every diet worldwide. Seeds, as well as many other parts of the plant, are also edible. Among legumes, soybeans are an important source of isoflavones, containing high concentrations of daidzein and genistein [32,37,38]. Daidzein and genistein are responsible for many of the health benefits of soy [39,40]. The amount of soy isoflavone in diets worldwide has increased because it is associated with a reduced risk of certain types of cancers, cardiovascular problems, osteoporosis, and menopausal symptoms [24,41,42,43,44]. In the present study, the leaves of five soybean cultivars were irradiated with UV-C (260 nm) and their effect on the concentrations of isoflavones was determined. Isoflavone compounds were extracted from the leaves and quantified using liquid chromatography-mass spectrometry (LC–MS).

## 2. Results

### 2.1. Effect of UV-C on Isoflavone Concentrations in Different Cultivars of Soybean

**Figure 2:**
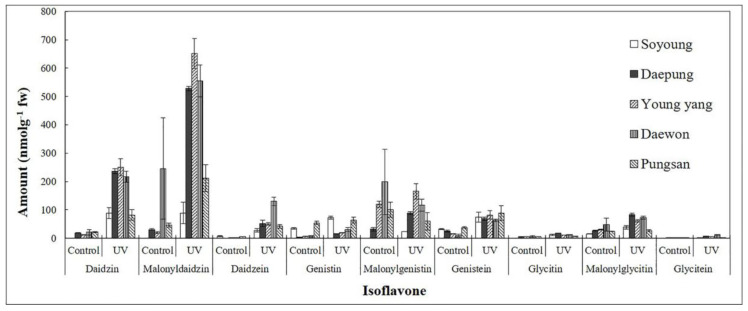
Isoflavone concentrations in five different cultivars of soybean leaves after 5 min UV-C treatment. The *x*-axis represents the different isoflavone compounds in five different cultivars and the *y*-axis represents the concentration of isoflavone compounds (unit: nmol g^−1^ of fresh weight). The error bars represent the means ± standard deviation (*n* = 3).

UV-C irradiation of soybean leaves has been associated with higher concentrations of isoflavone compounds. Nine isoflavone compounds, namely, daidzin, malonyldaidzin, and daidzein at mass-to-charge ratio: *m*/*z* 255; genistin, malonylgenistin, and genistein at *m*/*z* 271; and glycitin, malonylglycitin, and glycitein at *m*/*z* 285 were identified by LC–MS analysis. The concentrations of different isoflavonoid compounds and total isoflavone in the leaves of five cultivars of *G. max* after UV-C treatment are shown in Table S1 and Figure 2.

**Figure 3:**
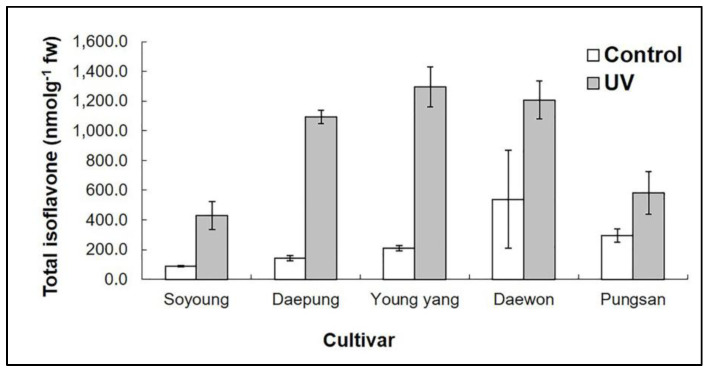
Total isoflavone concentration in different cultivars after 5 min UV-C treatment. The *x*-axis represents the different cultivars of soybean, whereas the *y*-axis represents the total isoflavone concentration (unit: nmol g^−1^ of fresh weight). The error bars represent the means ± standard deviation (*n* = 3). Means with the same letter are not significantly different (*p* ≤ 0.05).

The total isoflavone concentrations were also higher after 5 min UV-C treatment compared with the controls. The total isoflavone concentrations after UV-C treatment were 1094 and 1296.57 nmol g^−1^ of fresh weight in the cultivars Daepung and Young-yang, respectively (Table S1, Figure 3). Compared with the control (without UV-C irradiation), isoflavone concentration in irradiated leaves was 7.72- and 6.18-fold higher in the cultivars Daepung and Young-yang, respectively. We observed a large variation in isoflavone concentration among the different cultivars. Hence, selection of cultivars is a significant parameter for optimizing the isoflavone concentration in soybean [45].

Aglycone compounds were observed in different cultivars of soybean after 5 min irradiation. The concentrations of daidzein and glycitein were 130.21 and 11.73 nmol g^−1^ of fresh weight, respectively, in the cultivar Daewon. Similarly, genistein concentration was 81.88 nmol g^−1^ of fresh weight in the cultivar Young-yang (details are given in Table S1).

### 2.2. Effect of UV-C on Isoflavone Concentration in G. max cv. Daepung

**Figure 4:**
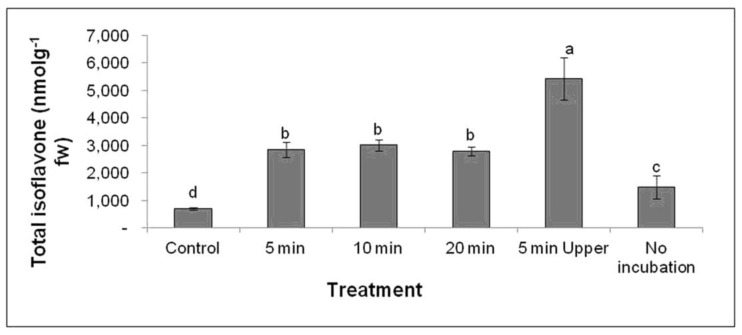
Total isoflavone concentration in *Glycine max* cv. Daepung after UV-C treatment for different time periods (unit: nmol g^−1^ of fresh weight). Here, leaves without UV-C irradiation and incubated in the dark are considered as the control. The leaves without UV-C irradiation as well as without incubation are indicated as “no incubation”. The leaves in which adaxial (upper) surface facing the UV light source, is termed as “5 min Upper”. The error bars represent the means ± standard deviation (*n* = 3). Means with the same letter are not significantly different (*p* ≤ 0.05).

After determining the most effective cultivar for total isoflavone induction under UV-C irradiation, we investigated the appropriate time period for UV-C irradiation. The UV-C treatment of soybean leaves was conducted for different lengths of time with the cultivar Daepung, and isoflavone concentration was estimated using LC–MS (Table S2 and Figure 4). The highest total isoflavone concentration, 5430 nmol g^−1^ of fresh weight, was observed in the treatment where the adaxial (upper) surface was facing the UV-C source for 5 min (Figure 4). This treatment resulted in a 7.75-fold higher total isoflavone concentration than that in the control.

**Figure 5:**
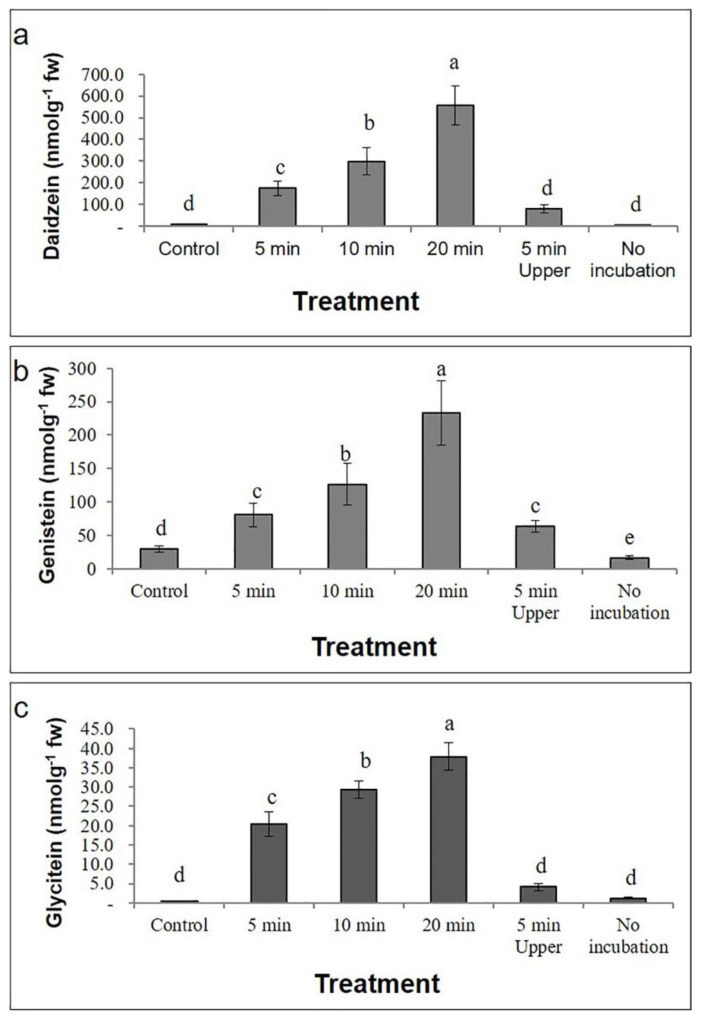
Concentrations of the aglycone compounds (**a**) daidzein, (**b**) genistein, and (**c**) glycitein in *G. max* cv. Daepung after UV-C irradiation for different time periods (unit: nmol g^−1^ of fresh weight). The treatment without UV-C irradiation and without incubation is indicated as “no incubation”. The error bars represent the means ± standard deviation (*n* = 3). Means with the same letter are not significantly different (*p* ≤ 0.05).

**Figure 6:**
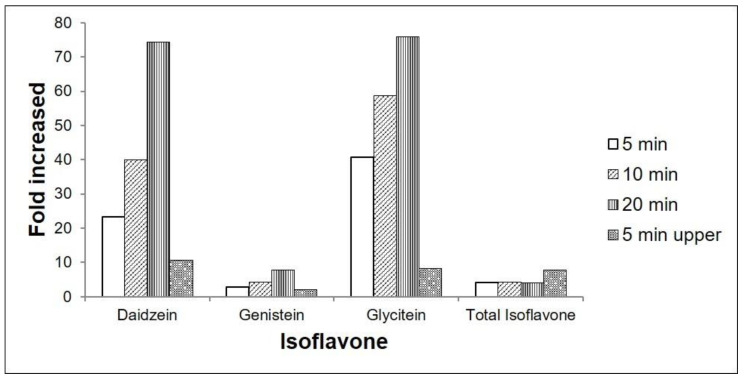
Approximate fold increase in aglycone compounds (daidzein, genistein, and glycitein) and total isoflavone concentration in *G. max* cv. Daepung after UV-C irradiation. Bars represent the fold increase after UV-C treatment for different time periods compared to that of the control.

The highest concentrations of the glycoside compounds daidzin, malonyldaidzin, genistin, malonylgenistin, glycitin, and malonylglycitin were 984.3, 2572, 397.3, 981.7, 39.1, and 307.3 nmol g^−1^ of fresh weight, respectively, and these were observed after the adaxial (upper) surface was facing the UV-C source for 5 min (Table S2). Conversely, the highest concentrations of the aglycone compounds daidzein, genistein, and glycitein were 557.9, 233.3, and 37.9 nmol g^−1^ of fresh weight, respectively, and these were observed after 20 min UV-C treatment (Table S2, Figure 5). The approximate fold increases in aglycone compound concentration (as compared to that of the control) for each treatment are presented in Figure 6.

## 3. Discussion

Isoflavones are phytochemicals present in legumes and are predominately found in soybean. Soybean plants contain a relatively high amount of isoflavone compounds compared with other plants. Since they are a rich source isoflavones, they are often included in the human diet [46]. Given that abiotic and biotic stresses can increase the concentrations of many metabolic compounds in plants, it is possible that this can also be the case for isoflavones in soybean plants [2,47,48,49,50,51]. Therefore, we investigated the low dose of UV-C could act as an elicitor for the induction of isoflavones in soybean plants. We estimated the concentrations of nine isoflavones (six glucosides and three aglycones) under various doses of UV-C irradiation.

After 5 min UV-C irradiation, the total isoflavone concentrations in cultivars Daepung and Young-yang were 7.72- and 6.18-fold higher, respectively, than those in the control. After irradiation, the malonyldaidzin concentration in the cultivar Young-yang was 33.41-fold higher than that in the control (Figure 3, Table S1). After 5 min UV-C treatment, the highest concentrations of malonyldaidzin and malonylgenistin (651.91 and 165.05 nmol g^−1^ of fresh weight, respectively) were observed in the cultivar Young-yang, and the highest concentration of malonylglycitin (83.18 nmol g^−1^ of fresh weight) was observed in the cultivar Daepung. The concentrations of malonyl glucosides, a major form of glucosides, increased in response to 5 min UV-C irradiation. Malonyl glucosides are thermally unstable and convert into their corresponding glycosides [30,52]. Previously, it has been reported that 1 mM and 100 µM salicylic acid applied at the blooming stage of soybean increased malonyldaidzin concentration by 18% and 15.5%, respectively [53]. In a previous study, genistin, genistein, daidzein, and biochanin-A concentrations were increased significantly after UV irradiation in callus cultures of *Genista tinctoria*. Genistin concentration increased by approximately 3% after 5 min UV 254 nm radiation and by 2% after 2 min UV 366 nm radiation [53].

In general, the concentrations of aglycone isoflavones are in the order genistein > daidzein > glycitein, with concentrations of each compound depending on the cultivar and local environmental conditions. For example, hot and dry weather can result in low-grade crops, which lead to a reduction in overall isoflavone concentration [54]. Daidzein is the aglycone of daidzin and malonyldaidzin, genistein is the aglycone of genistin and malonylgenistin, and glycitein is the aglycone of glycitin and malonylglycitin. Replacement of the sugar residue (glycosyl group) with a hydrogen atom leads to the conversion of glycosides to aglycone compounds. Our study revealed that after 5 min UV-C treatment, the concentrations of the aglycone compounds daidzein, glycitein, and genistein were increased significantly many fold in all five soybean cultivars (Table S1). 

In a previous study, 1 mM salicylic acid applied at the full pod stage increased glycitein concentration by 81% [55]. Similarly, in another study, the total isoflavone concentration in soybean seeds was 92.7% higher than that in a control after application of 0.1 mM salicylic acid. Daidzein and glycitein concentrations in soybean seeds were found to increase by 53.7% and 78.7%, respectively, while genistein concentration only increased by 2.9% after the application of 0.5 mM methyl jasmonate in soybean seeds compared with a control [56]. Similarly, following the application of 1 mM ethyl acetate, glycitein concentration increased by 3.3-fold; however, daidzein and genistein concentrations only increased by 12.3% and 10.2%, respectively, at the full pod stage of soybean [55]. Light illumination positively affects the callus induction in three Korean cultivars of soybean. In a study where the isoflavone synthase gene was transformed to promote higher isoflavone concentrations, the total isoflavone concentration in the callus tissues of the transformed line was three- to sixfold higher than that in the control cell line [57]. In another study, NaN_3_-induced soybean mutants had higher total isoflavone concentrations (on average 5% to 25% higher) than their wild-type cultivar [58]. Furthermore, many studies have demonstrated that postharvest UV-C treatment in detached leaves, vegetables and fruits increases the secondary metabolite contents and their biosynthetic enzymes [59,60,61,62,63,64].

The majority of aglycone isoflavones are in the free and conjugated forms of genistein and daidzein, which constitute 60% and 30% of the total aglycone isoflavone concentration, respectively, while the glycitein group is a minor component (10%) [65,66]. Aglycones are generated mainly from malonyl glucosides [30,52], which are possibly converted into aglycones in response to UV-C. This would explain the high aglycone concentrations in our experiment. It has also been reported that glycitein is responsive to elicitors such as salicylic acid, methyl salicylate, and ethyl acetate; aglycone concentrations were 256% higher in soybean plants following 1 mM ethyl acetate treatment than under a control treatment. In an isoflavone synthase-transformed soybean cv. Sojin cell line, there were comparatively higher concentrations of malonyldaidzin than that of malonylgenistin, genistein, and daidzein. However, the concentrations of malonylgenistin, daidzein, and genistein were similar to those found in the non-transformed cell line of soybean [57].

UV-C irradiation can influence the photosynthetic pigments and other physiological and biochemical components of plants [3,4,16,67]. It has been found that the concentrations of carotenoids, anthocyanins, flavonoids, and proline were significantly higher following UV-C treatment compared with a control [16,63,67,68,69,70,71]. The pigment apigeninidin (3-deoxy anthocyanidin) accumulated in the epidermal layer of soybean cotyledons irradiated for 60 min with UV-C radiation. Interestingly, the authors said that this pigment was not verified in soybean species primarily, and only UV-C led to its induced accumulation. An in vitro test showed that apigeninidin had the ability to reduce lipid radicals in a dose-dependent manner and possessed antioxidant activities [51]. UV-C irradiation plays an important role in some biological processes such as cell rescue, protein fate, secondary metabolism, and transcription regulation [64]. UV-C treatment can increase the antioxidant activity in fresh cut red cabbage in storage [63,72], and can increase the trans-resveratrol concentration from 2.19 to 56.76 µg g^−1^ of fresh weight in *Vitis amurensis* cv. Tonghua-3. The increase in concentration of secondary metabolites may be linked to the transcriptional regulation of genes involved their biosynthesis and signaling [73,74,75,76]. Thus, UV-C irradiation has been directly correlated with the regulation of key biosynthetic enzymes such as stilbene synthase (STS), phenylalanine ammonia lyase, and chalcone synthase (CHS), etc.

Higher isoflavone concentrations in soybean leaves were observed in the present study after UV-C irradiation from a 30 cm distance. This may have been due to higher activities of the major biosynthetic enzymes such as chalcone synthase (CHS), chalcone reductase (CHR), and isoflavone synthase (IFS), since these enzymes may be significantly induced after UV elicitation [77,78]. Thus, low doses of UV-C allow the plants to partially recover to their normal physiological status and trigger the key enzymes of their metabolic pathway, ultimately leading to the production isoflavone compounds in soybean leaves.

## 4. Materials and Methods

### 4.1. Plant Materials

Soybean seeds of five cultivars, namely, Soyoung, Daepung, Young-yang, Daewon, and Pungsan, were sown in pots containing sand and soil (1:1). Plants were grown in a greenhouse at normal conditions (20 ± 2 °C temperature and 16 light/8 darkness hour photoperiod) and watered regularly. When the plants were 6 weeks old, we collected healthy and morphologically similar leaves from the same plant for further experimentation.

### 4.2. Ultraviolet-C Irradiation

For the first experiment, the leaves of each cultivar collected from the greenhouse were placed in separate vinyl zipper polybags, weighed individually, and immediately transferred to a plastic box containing a wet paper towel. Leaves were exposed to UV-C (260 nm) from 30 cm distance at 24 °C in the dark. The abaxial (lower) surface faced the source of light for different time periods. Further, one experimental leaf sample was placed with the adaxial (upper) surface facing the light. After UV-C exposure, the leaves were incubated in a growth chamber at 24 °C in the dark for 24 h. The controls consisted of leaves that underwent the same treatment lacking the UV-C exposure. After incubation, the leaves were stored separately in individual vinyl zipper bags for 24 h in a deep freezer (−80 °C) until isoflavone extraction. For the second experiment, morphologically similar leaves of the cultivar Daepung were collected and exposed to UV-C radiation for three different periods of time (5, 10, and 20 min). As in the first experiment, leaves were then incubated and frozen for 24 h. Similar to the first experiment, in the second experiment, the control consisted of leaves that underwent the same treatment lacking the UV-C exposure. Additionally, the leaves without UV-C irradiation as well as without incubation (termed as no incubation) were also included.

### 4.3. Preparation of Plant Extracts

The previously frozen leaf samples were ground to a powder using a mortar and pestle and 80% ethanol (9 mL ethanol for 1 g of leaf). The samples were then placed in 50 mL falcon tubes and shaken for 1 h at room temperature in the dark. Samples were then filtered using a nylon filter (Nylon 0.22 µm), and 1.8 mL of filtrate was collected for LC–MS analysis.

### 4.4. Quantification of Isoflavonoids by LC–MS Analysis

The concentrations of isoflavone compounds were determined using an LC–MS system consisting of a high-performance liquid chromatography (HPLC) apparatus (model 2695; Waters, Milford, MA, USA) equipped with a pentafluorophenyl column (Luna C18 reversed phase column, 4.6 × 150 mm, 5 µm; Phenomenex, Torrance, CA, USA) and a mass spectrometer (model 3100; Waters). The conditions for HPLC were as follows: injection volume = 5 µL; solvent A = 20 mM ammonium acetate buffer (pH adjusted to 7.2); solvent B = acetonitrile; and solvent program = 50:50 (A/B) to 50:50 (A/B) over 10 min at a flow rate of 0.5 mL min^−1^. The mass spectrometry analysis conditions were as follows: N_2_ gas flow rate = 4 L h^−1^; temperature = 350 °C; capillary voltage = 4 kV; cone voltage = 30 V; ionization mode = electrospray positive; single ion recording (*m*/*z*) = 255 for daidzin, malonyldaidzin, and daidzein; *m*/*z* = 271 for genistin, malonylgenistin, and genistein; and *m*/*z* = 285 for glycitin, malonylglycitin, and glycitein.

### 4.5. Statistical Analysis

The graphs and tables were prepared using Microsoft Office Excel. All data are expressed as the mean ± standard deviation (SD) from three independent replicates in each experiment. Statistical analysis of samples was conducted using one-way analyses of variance (ANOVAs) followed by Duncan’s multiple range tests at a *p*-value of <0.05 using Statistical Analysis System (SAS version 9.4, SAS Institute Inc., Cary, NC, USA).

## 5. Conclusions

Isoflavones have a chemical structure similar to that of human estrogen. They play a significant role in a healthy diet and help in fighting disease. Soybean plants produce three groups of isoflavones, which belong to four chemical forms: aglycones (daidzein, genistein, and glycitein), β-glucosides (daidzin, genistin, and glycitin), acetylglucosides (acetyldaidzin, acetylgenistin, and acetylglycitin), and malonylglucosides (malonyldaidzin, malonylgenistin, and malonylglycitin), making a total of 12 isoflavones. In the present study, nine isoflavone compounds (all forms except acetylglucosides) were detected in soybean leaves following UV-C treatment (5 min and 30 cm from the leaf surface). The large variation in isoflavone concentrations among different cultivars suggested that cultivar selection is a key parameter for optimizing the isoflavone concentration. Among all isoflavones, the most beneficial are the aglycosides, especially genistein and daidzein, which are important for maintaining good bone density, and their concentrations were significantly higher after UV elicitation. In the current study, concentrations of aglycone isoflavones in the cultivar Soyoung after 5 min UV-C irradiation followed the order genistein > daidzein > glycitein, but in all other cultivars, it followed the order daidzein > genistein > glycitein.

On the basis of the results, the cultivar Daepung was selected as the most effective cultivar for isoflavone induction through UV-C irradiation. Then, the optimal irradiation length for aglycoside and glycoside isoflavone compound production was determined. We found that aglycoside compound concentrations were the highest after 20 min UV-C irradiation, while glycoside compound concentrations were the highest after 5 min of the adaxial surface facing the UV-C source. Overall, this study shows that both the cultivar and length of UV-C exposure influence isoflavone concentrations in soybean plants. However, the exact mechanism of isoflavone induction through UV-C elicitation is still unexplored and requires further experimental validation.

## Supplementary Materials

The following are available online at https://www.mdpi.com/2223-7747/9/8/1043/s1, Table S1: Effect of UV-C (260 nm, 5 min) irradiation on isoflavone concentrations in different cultivars of *Glycine max*. Table S2: Isoflavone concentrations in *Glycine max* (cv. Daepung) after UV-C (260 nm) irradiation.
