# Supplementary material for: Effect of Ultraviolet C Irradiation on Isoflavone Concentrations in Different Cultivars of Soybean (Glycine max)
Source: Plants (Basel). 2020 Aug 16;9(8):1043. doi: 10.3390/plants9081043 (PMC7464170; doi:10.3390/plants9081043)
Supplement: Supplementary file 1 [file plants-09-01043-s001.pdf]

plants

MDPI

## Supplementary Materials

**Table S1.** Effect of UV-C (260 nm, 5 min) irradiation on isoflavone concentrations in different cultivars of *Glycine max*Data represent the means of triplicate measurements ± standard deviation. Unit: nmol g-1 of fresh weight.

| Cultivars | Treatment | Daidzein | M-daidzin | Daidzin | Genistein | M-genistin | Genistin | Glycitein | M-glycitin | Glycitin | Total Isoflavone |
|---|---|---|---|---|---|---|---|---|---|---|---|
| Soyoung | Control | 7.40±1.3 | 0 | 0 | 31.29±1.9 | 0 | 34.97±2.4 | 0 | 15.14±0.2 | 0 | 88.80±5.7 |
| | UV-C | 27.54±5.78 | 89.0±38.4 | 88.52±18.7 | 74.82±17.4 | 23.08±0 | 72.43±5.0 | 2.21±0.21 | 38.71±6.4 | 11.78±2.3 | 428.09±94.2 |
| Daepung | Control | 0 | 30.65±3.0 | 18.7±1.6 | 25.0±3.6 | 31.16±6.9 | 4.02±0.2 | 0.43±05 | 26.61±1.6 | 5.22±0.3 | 141.80±17.1 |
| | UV-C | 52.19±10.7 | 527.67±7.9 | 235.62±8.5 | 68.135.8 | 87.50±5.3 | 15.61±1.3 | 7.02±0.7 | 83.18±5.9 | 17.83±0.8 | 1094±46.7 |
| Young-yang | Control | 1.25±0.2 | 19.51±3.9 | 12.05±0.5 | 15.23±1.6 | 119.57±10.6 | 6.20±0.6 | 0.60±0.2 | 29.89±1.5 | 5.42±0.1 | 209.73±19.0 |
| | UV-C | 49.95±4.7 | 651.91±53.0 | 250.81±29.7 | 81.88±14.4 | 165.05±27.6 | 19.09±0.6 | 5.54±0.75 | 61.58±4.6 | 10.77±0.7 | 1296.57±135.9 |
| Daewon | Control | 1.74±0 | 245.22±178.5 | 20.55±9.1 | 9.69±5.2 | 199.03±115.5 | 6.66±2.1 | 1.16±0.2 | 48.22±22.5 | 6.30±2.0 | 538.57±335.1 |
| | UV-C | 130.21±15.0 | 554.4±56.6 | 216.99±18.7 | 62.85±4.4 | 116.27±20.9 | 30.22±6.3 | 11.73±0.1 | 72.54±5.1 | 12.33±1.0 | 1207.54±128.1 |
| Pungsan | Control | 5.71±0.6 | 45.83±7.51 | 21.56±1.5 | 37.96±3.0 | 100.93±26.2 | 53.83±6.44 | 0.42±0.0 | 23.36±1.1 | 5.27±0.1 | 294.87±46.3 |
| | UV-C | 42.1±6.2 | 211.2±47.5 | 82.07±18.74 | 88.50±27.0 | 59.26±31.4 | 64.34±10.6 | 1.07±0.1 | 27.17±3.8 | 6.51±0.7 | 582.20±146.0 |

**Table S2.** Isoflavone concentrations in *Glycine max* (cv. Daepung) after UV-C (260 nm) irradiation.

| Table . | Daidzin | M-daidzin | Daidzein | Genistin | M-genistin | Genistein | Glycitin | M-glycitin | Glycitein | Total isoflavone |
|---|---|---|---|---|---|---|---|---|---|---|
| Control | 32.7±4.3 | 237.4±23.1 | 7.5±0.9 | 54.5±6.7 | 275.7±21.7 | 29.8±4.4 | 4.7±0.6 | 57.6±10.6 | 0.5±0.2 | 700±52.8 |
| 5 min | 418.9±36.7 | 1277.2±118.1 | 175.5±34.0 | 124.2±10.6 | 420.4±51.0 | 80.9±17.4 | 34.4±2.5 | 286.4±12.4 | 20.4±3.2 | 2838±282.2 |
| 10 min | 427.6±29.8 | 1188.5±106.7 | 299.2±63.2 | 149.1±18.8 | 448.5±31.3 | 126.4±30.9 | 37.8±2.6 | 298.9±12.6 | 29.4±2.2 | 3005±192.5 |
| 20 min | 263.9±19.4 | 840.7±31.6 | 557.9±89.4 | 129.8±15.7 | 477.9±43.0 | 233.2±47.6 | 22.8±4.9 | 224.2±13.1 | 37.9±3.5 | 2788±157.6 |
| 5 min Upper | 984.3±90.3 | 2572.5±268.7 | 80.3±18.4 | 397.3±157.5 | 981.7±251.4 | 63.8±8.4 | 39.1±4.5 | 307.3±14.8 | 4.1±1.0 | 5430±775.5 |
| No incubation | 65±19.2 | 553.8±176.6 | 4.5±0.7 | 58.5±14.5 | 573.2±143.2 | 16.7±2.8 | 16.8±3.4 | 191.4±73.8 | 1.2±0.3 | 1481±429.7 |
